# The Natural Antimicrobial *trans*-Cinnamaldehyde Interferes with UDP-N-Acetylglucosamine Biosynthesis and Cell Wall Homeostasis in *Listeria monocytogenes*

**DOI:** 10.3390/foods10071666

**Published:** 2021-07-20

**Authors:** Lei Sun, Gil Rogiers, Chris W. Michiels

**Affiliations:** Department of Microbial and Molecular Systems, KU Leuven, B-3000 Leuven, Belgium; lei.sun@kuleuven.be (L.S.); gil.rogiers@milcobel.com (G.R.)

**Keywords:** UDP-GlcNAc biosynthesis, YvcK, *Listeria monocytogenes*, natural antimicrobial, *trans*-cinnamaldehyde, food preservative

## Abstract

*Trans*-cinnamaldehyde (*t*-CIN), an antimicrobial compound from cinnamon essential oil, is of interest because it inhibits various foodborne pathogens. In the present work, we investigated the antimicrobial mechanisms of *t*-CIN in *Listeria monocytogenes* using a previously isolated *yvcK::Himar1* transposon mutant which shows hypersensitivity to *t*-CIN. Time-lapse microscopy revealed that *t*-CIN induces a bulging cell shape followed by lysis in the mutant. Complementation with wild-type *yvcK* gene completely restored the tolerance of *yvcK::Himar1* strain to *t*-CIN and the cell morphology. Suppressor mutants which partially reversed the *t*-CIN sensitivity of the *yvcK::Himar1* mutant were isolated from evolutionary experiments. Three out of five suppression mutations were in the *glmU-prs* operon and in *nagR*, which are linked to the biosynthesis of the peptidoglycan precursor uridine-diphosphate-N-acetylglucosamine (UDP-GlcNAc). GlmU catalyzes the last two steps of UDP-GlcNAc biosynthesis and NagR represses the uptake and utilization of N-acetylglucosamine. Feeding N-acetylglucosamine or increasing the production of UDP-GlcNAc synthetic enzymes fully or partially restored the *t*-CIN tolerance of the *yvcK* mutant. Together, these results suggest that YvcK plays a pivotal role in diverting substrates to UDP-GlcNAc biosynthesis in *L. monocytogenes* and that *t*-CIN interferes with this pathway, leading to a peptidoglycan synthesis defect.

## 1. Introduction

*Listeria monocytogenes* is a Gram-positive foodborne pathogen that causes severe invasive listeriosis and meningitis among susceptible persons such as immunocompromised individuals, pregnant women and elderly persons [1,2]. It is a versatile and resilient organism that thrives well in a wide range of natural and human-made environments, including soil, freshwater, decaying plant material, and the gastrointestinal tract of various animals [3]. It can also establish in the resident house microbiota of food production facilities, and as such it is a common contaminant during food production and storage [2]. Furthermore, *L. monocytogenes* is highly salt tolerant and can multiply in foods at temperatures as low as 0 °C [4].

Because of its widespread occurrence, contamination of foods with *L. monocytogenes* cannot always be prevented, and preservatives are used in some foods to prevent outgrowth of the pathogen to high numbers [4,5]. Despite their effectiveness, traditional preservatives are increasingly under scrutiny for possible adverse health effects, and food producers are exploring more natural alternatives to replace them. Plant essential oils have received much attention in this respect, since they constitute an immense reservoir of compounds that are active against a wide range of microorganisms [5]. A well-studied compound is *trans*-cinnamaldehyde (*t*-CIN), one of the major components of cinnamon essential oil [6]. The most notable structural feature of *t*-CIN is the presence of an α,β-unsaturated aldehyde functional group, which confers electrophilic and thiol-reactive properties to the compound [6]. Our previous analysis showed that growth inhibition of *L. monocytogenes* by *t*-CIN is typically characterized by elongation of the lag phase [7]. Several studies have addressed the antimicrobial mechanisms of *t*-CIN and multiple hypotheses have been proposed including the inhibition of cell division [8], alteration of cell membrane composition and permeability [9] and reduction in intracellular ATP levels [10]. However, these effects are quite general secondary effects and precise insight in the primary cellular targets are not provided; hence, more specific approaches are therefore necessary.

In our previous work, a genome-wide random *Himar1* transposon mutant library was constructed in *L. monocytogenes* Scott A, and one of the mutants showing increased *t*-CIN sensitivity had a transposon insertion in the *yvcK* gene (*yvcK::Himar1*) [7]. However, the precise function of YvcK in *L. monocytogenes* was obscure. Loss of YvcK increased the sensitivity of *L. monocytogenes* to lysozyme and cell wall targeting antibiotics such as ampicillin, bacitracin and ceftriaxone, and caused severe growth and morphology defects when growing the bacteria in minimal media with glycerol as the primary carbon source [11]. Similarly, deletion of *yvcK* in *Bacillus subtilis* and *Mycobacterium smegmatis* induced deformed cell shape, attenuated growth on non-glycolytic carbon sources, and an elevated sensitivity to cell wall targeting antibiotics [12,13,14]. The similar phenotypes caused by loss-of-function mutations suggest a conserved function of YvcK in cell wall integrity and optimal carbon utilization in a broad range of Gram-positive bacteria, but its detailed cellular function needs to be further clarified. 

A study of suppression mutations which restored either the attenuated growth on gluconeogenic carbon sources or the sensitivity to cefuroxime of a *∆yvcK* mutant was recently conducted in *B. subtilis* [13]. Several mutations induced elevated expression of glucosamine-6-phosphate synthase (GlmS) and phosphoglucosamine mutase (GlmM), two key enzymes of the uridine-diphosphate-N-acetylglucosamine (UDP-GlcNAc) biosynthesis pathway [15]. Moreover, supplementation of N-acetylglucosamine (GlcNAc) to growth medium with a non-glycolytic carbon source alleviated the attenuated morphology of a *B. subtilis yvcK* null mutant [13]. Furthermore, YvcK has been demonstrated to stimulate the activity of GlmS, depending on the intracellular concentration of UDP-GlcNAc [13,16]. Therefore, YvcK in *B. subtilis* was suggested to play a role in diverting carbon sources from central metabolism to the synthesis of UDP-GlcNAc [13,16].

In this work, we have characterized the *t*-CIN hypersensitive transposon mutant *yvcK::Himar1* to generate a deeper insight into the mode of action of *t*-CIN in *L. monocytogenes*. The mutant showed cell shape deformations which were exacerbated in the presence of *t*-CIN. Suppressor mutants of *yvcK::Himar1* with partially reversed *t*-CIN sensitivity were isolated and had mutations residing in the promoter region of the *glmU-prs* operon and in the coding region of *nagR*, which are both connected to UDP-GlcNAc biosynthesis. Overproduction of UDP-GlcNAc biosynthetic enzymes [GlmU (bifunctional glucosamine-1-phosphate acetyltransferase/GlcNAc-1-phosphate uridyltransferase), GlmS and GlmM] and supplementation of GlcNAc to the growth medium restored at least partially the tolerance of the *yvcK* mutant to *t*-CIN. These findings suggest that YvcK plays an pivotal role in UDP-GlcNAc biosynthesis in *L. monocytogenes*, similar to its role in *B. subtilis* [13,16], and that *t*-CIN interferes with the UDP-GlcNAc homeostasis, probably by limiting the availability of the substrate Fru-6-P. 

## 2. Materials and Methods

### 2.1. Bacterial Strains and Growth Conditions

Bacterial strains and plasmids used in this work are listed in Table 1. *L. monocytogenes* Scott A was used as the wild-type (WT) strain and acquired from the International Life Sciences Institute (ILSI) North America [17]. *E. coli* DH5α [18] and S17-1 λpir [19] were employed as the host for cloning constructs and as donor strain for conjugational plasmid transfer, respectively. *L. monocytogenes* strains were grown at 30 °C in Brain Heart Infusion (BHI; Oxoid, Hampshire, UK). *E. coli* strains were grown in Luria-Bertani (LB; 10 g/L tryptone, 5 g/L yeast extract, 5 g/L NaCl) at 37 °C. Antibiotics were used when appropriate in the following concentrations: 50 µg/mL erythromycin (Acros Organics, Fair Lawn, NJ, USA) (Ery), 50 µg/mL kanamycin (AppliChem GmbH, Darmstadt, Germany) (Km), 100 μg/mL ampicillin (Thermo Fisher Scientific, Waltham, MA, USA) (Amp), 20 µg/mL polymyxin B sulfate (AppliChem GmbH) and 10 μg/mL chloramphenicol (Acros Organics) (Cm). Other chemicals used in this work include *t*-CIN (Acros Organics), N-Acetylglucosamine (Sigma-Aldrich, Saint Louis, MO, USA) and isopropyl β-D-1-thiogalactopyranoside (Acros Organics) (IPTG, 1 mM).

### 2.2. Evolutionary Study to Isolate yvcK::Himar1 Suppression Mutants with Regained t-CIN Tolerance

**Figure 1:**
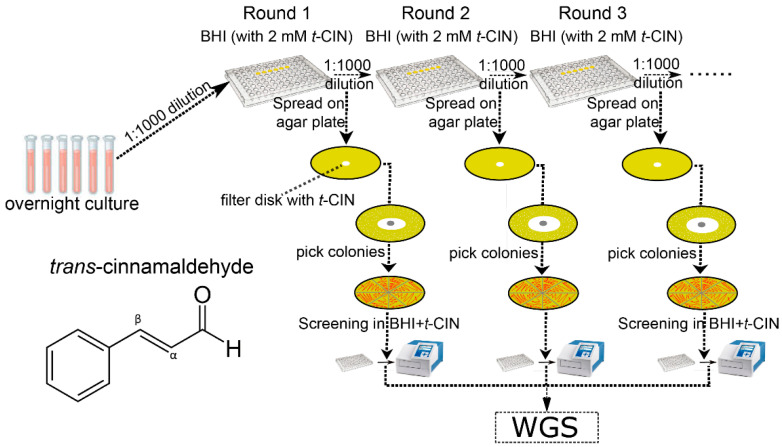
Scheme of the evolutionary experiment setup with *yvcK::Himar1* in BHI supplemented with 2 mM *t*-CIN as described in Materials and Methods. Isolates which recovered *t*-CIN tolerance were identified in all six independent lineages with *t*-CIN supplementation after three rounds of subculture, while no recovery of *t*-CIN tolerance was observed in control cultures without *t*-CIN. The molecular structure of *t*-CIN is also shown.

An experimental evolution experiment was conducted as illustrated in Figure 1. Independent colonies of the *yvcK::Himar1* strain were inoculated in six parallel test tubes with 4 mL BHI broth. After overnight incubation at 30 °C with shaking (250 rpm), the cultures were diluted 1000-fold in BHI containing 2 mM *t*-CIN. A culture in BHI without *t*-CIN, to which only the equivalent amount of ethanol added, was included as control without selection pressure. Two hundred µL portions of diluted cultures were transferred into a 96-well microplate, covered with a transparent adhesive foil (Greiner Bio-One EASYseal™ Adhesive Microplate Sealer, Thermo Fisher Scientific) to protect against evaporation and contamination, and incubated at 30 °C with continuous shaking (250 rpm) to reach a turbidity of OD630 ~0.7, determined with an automated Multiskan^TM^ FC microplate reader (Thermo Fisher Scientific), corresponding to stationary phase. Cultures were then again diluted 1:1000 in the same medium and passed to a fresh microplate for another round of growth. In each round, a portion of the stationary cultures was diluted 10^5^ fold and 100 µL was spread on BHI agar. A 6 mm sterile Whatman^®^ filter paper disc impregnated with 10 µL pure *t*-CIN was then placed in the center of the agar plate. An inhibition halo was formed around the paper disc after incubating the plate at 30 °C for two days and 16 colonies near the inhibition zone were streaked. The resistance of the isolates against 2 mM *t*-CIN was evaluated by a growth assay in the microplate reader. The evolution experiment was continued until isolates with (partially) restored *t*-CIN tolerance emerged. A selection of these isolates from independent cultures were sent for whole genome sequencing (WGS) to analyze mutations.

### 2.3. Whole Genome Sequencing

Genomic DNA was extracted from overnight cultures of *L. monocytogenes* with the GeneJET Genomic DNA purification kit (Thermo Fisher Scientific). The quality and concentration of genomic DNA was determined by gel electrophoresis, NanoDrop™ photometric and Qubit fluorometric analysis (Thermo Fisher Scientific). Paired-end libraries were constructed with the NEBNext Ultra DNA Library Prep Kit (NEB, Ipswich, MA, USA) and sequenced at VIB Nucleomics Core (Leuven, Belgium) with an Illumina MiSeq sequencer (Illumina, San Diego, CA, USA). Reads were analysed with CLC Genomic Workbench software (QIAGEN, Hilden, Germany) to determine mutations in the evolved strains compared to the *yvcK::Himar1* parental strain. All detected mutations were subsequently checked by targeted amplification and Sanger sequencing (Macrogen Europe, Amsterdam, The Netherlands).

### 2.4. Growth Assay

Growth curves were established by turbidity measurement (OD620 or OD630) with an automated microplate reader (Multiskan Ascent^®^ or Multiskan^TM^ FC, Thermo Fisher Scientific). Firstly, the OD600 of overnight cultures was determined with an Ultrospec™ 10 Cell Density Meter (Biochrom, Cambridge, UK) and slightly adjusted by supplying additional BHI to obtain the same value (OD600 ≈ 2) for all the cultures within a single experiment. The suspensions were then diluted 1000-fold in BHI to which 1 mM IPTG and/or 2 or 3 mM *t*-CIN had been added if necessary. Then, 200 µL aliquots were transferred to a 96-well microplate, the plate was sealed with a transparent adhesive foil and incubated at 30 °C in an automated microplate reader. Every 15 or 30 min, the plate was shaken at 960 rpm and OD620 was recorded. The Excel add-in package DMFit (Quadram Institute Bioscience, Norwich, United Kingdom) was used to determine the maximum growth rate (µmax), the lag phase time (λ) and the maximal OD (ODmax) value at stationary phase through the Baranyi and Roberts model [20].

### 2.5. Microscopy and Cell Dimension Measurement

To measure cell dimensions, one µL of an appropriately diluted late exponential culture (OD_600_ ≈ 1) was applied to 2% agarose pads deposited on a microscopy slide on which a cover glass was mounted using a Gene Frame (Thermo Fisher Scientific). Observations were made with an Eclipse Ti-E inverted microscope (Nikon, Champigny-sur-Marne, France) equipped with a CoolSnap HQ2 FireWire CCD-camera. Images were acquired using NIS-elements software (Nikon), and cell width and length were determined with the MicrobeTracker image analysis software [24], with manual curation to remove false segmentation. For the time-lapse phase-contrast microscopy, the agarose pads were prepared with BHI supplemented with 1 mM *t*-CIN. Overnight stationary cultures were diluted 50-fold in BHI with 1 mM *t*-CIN and one µL diluted culture was applied to the BHI agarose pad. Observation was performed at temperature set at 30 °C and an image was acquired every 30 min for 24 h.

### 2.6. Genetic Complementation of Mutant Strains

For genetic complementation of the *yvcK::Himar1* mutant, the wild-type *yvcK* gene was amplified using primer pair yvcK_NcoI/yvcK_SalI (Table 2), cleaved with the restriction enzymes NcoI and SalI, and cloned in pIMK3 restricted with the same enzymes, using standard cloning procedures. After verification with Sanger sequencing, the construct was conjugated from *E. coli* S17-1 λpir into *L. monocytogenes yvcK::Himar1*. Successful chromosomal integration was confirmed via PCR with primers yvcK_NcoI and NC16(II) (Table 2) (which anneal left and right of the integration site and point inwards) and Sanger sequencing with pIMK_FW/pIMK_REV (Table 2) primer pair, which point towards the *yvcK* gene from both sides of the pIMK3 cloning site. The complementation strain was designated as *yvcK/pIMK3-yvcK*. Control strains were constructed by integration of the empty pIMK2 and pIMK3 plasmid into WT and *yvcK::Himar1* strains, respectively, and were designated as *WT/pIMK2* and *yvcK/pIMK3*, respectively. 

The same strategy was used to overexpress *glmU*, *glmM, glmS*, and the WT and mutant allele of *nagR* in *L. monocytogenes*, using glmU_NcoI/glmU_SalI, glmM_NcoI/glmM_SalI, glmS_NcoI/glmS_SalI and nagR_BspHI/nagR_SalI primer pairs, respectively (Table 2). The integration vectors used were pIMK2 (*glmM*, *glmS* and *nagR*) or pIMK3 (*glmU*) [21]. The plasmid constructs and strains are listed in Table 1.

### 2.7. Construction of nagR Deletion Mutant

The pKSV7-oriT plasmid was utilized to generate in-frame deletions of *nagR* as described [22,23]. Firstly, approximately 1 kb fragments from upstream and downstream of *nagR* were amplified with nagR-KO-A/B and nagR-KO-C/D primer pairs (Table 2). The obtained PCR products were diluted 100-fold, mixed in a ratio of 1:1 and employed as template for overlapping extension PCR utilizing nagR-KO-A/D primer pair. The obtained ~2 kb PCR fragment and pKSV7-oriT were then digested with KpnI and PstI restriction enzymes and ligated overnight. Following transformation to *E. coli* DH5α, constructs were extracted and checked by PCR and Sanger sequencing with pKSV7-CK-F and pKSV7-CK-R primers. The construct was then electro-transformed to *L. monocytogenes* as described [21]. After recovery in BHI broth at 30 °C for three hours, the cells were spread on BHI agar plates with Cm (10 μg/mL) and incubated at 30 °C for two days. Allelic exchange was achieved with a colony picked from the plate as previously described [23]. Mutants were identified by colony PCR with nagR-KO-A and nagR-KO-D primers and Sanger sequencing were then performed to identify a successful deletion mutant. 

### 2.8. Statistical Analysis 

Growth parameters calculated from the growth assay are presented as means ± standard deviation (SD) of three biological replicates. The significance of mean differences was calculated by the Tukey’s honestly significant difference (Tukey’s HSD) test using GraphPad PRISM 7.0 (GraphPad, San Diego, CA, USA). *p* values < 0.05 were considered statistically significant. 

## 3. Results

### 3.1. Characterization of the t-CIN Hypersensitive yvcK::Himar1 Mutant

**Figure 2:**
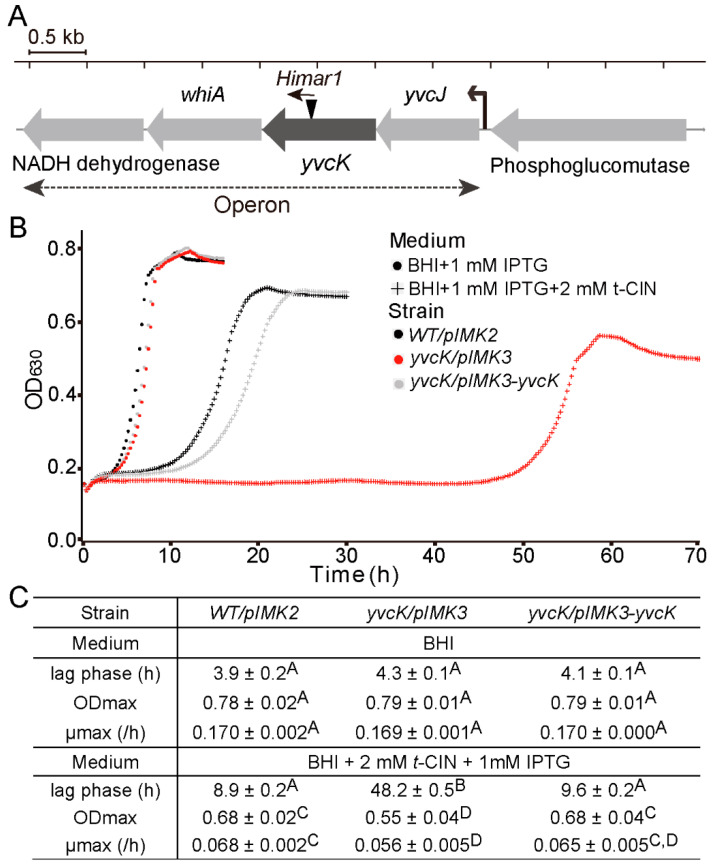
The transposon mutant *yvcK::Himar1* is hypersensitive to *t*-CIN. (**A**) The gene context of *yvcK* in the genome of *L. monocytogenes* Scott A, with the insertion of the *Himar1* transposon (black triangle) indicated. The arrow on top of the transposon indicates the orientation of the erythromycin resistance gene (ermC). Four genes, including *yvcJ*, *yvcK*, *whiA* and the gene predicted to encode an NADH dehydrogenase, form an operon [25]. A transcription start site is indicated with a black arrow. (**B**) Growth curves of *WT/pIMK2* (black), *yvcK/pIMK3* mutant (red) and the complemented strain *yvcK/pIMK3-yvcK* (grey) in BHI broth (dot) and in BHI broth with 2 mM *t*-CIN and 1 mM IPTG (cross). All curves represent mean values of three independent cultures. (**C**) Lag phases (λ), maximum growth rates (μmax) and maximum optical densities (ODmax) from the growth curves in (**B**) are listed in the table. Values are mean ± SD (*n* = 3) and those followed by a common letter are not significantly different at the 5% level of significance.

As aforementioned, a *t*-CIN hypersensitive mutant *yvcK::Himar1* was isolated in a screening of a *L. monocytogenes* Scott A transposon mutant library [7]. The *Himar1* transposon is inserted at 655 bp from the start codon (Figure 2A) and WGS analysis demonstrated no additional mutation. The *yvcK* gene is part of an operon comprising the ORFs *yvcJ*, *yvck*, *whiA* and a gene predicted to encode a NADH dehydrogenase [25] (Figure 2A). However, their intracellular functions are poorly characterized.

**Figure 3:**
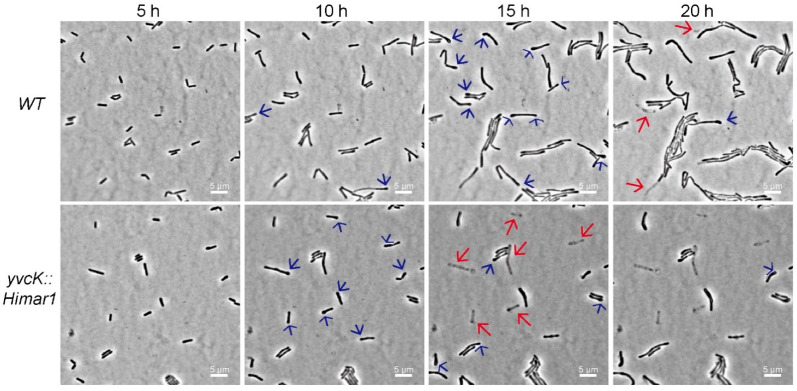
Time-lapse observation of WT and *yvcK::Himar1* in BHI supplemented with 1 mM *t*-CIN at 30 °C. Phase contrast images were acquired every 0.5 h and images of a representative specific field at 5 h, 10 h, 15 h and 20 h are presented. The red arrows indicate cell lysis of *yvcK::Himar1*. Polar bulging (blue arrows) can be observed at 10 h, 15 h and 20 h and is more pronounced for the *yvcK* mutant cells at 10 h.

**Figure 4:**
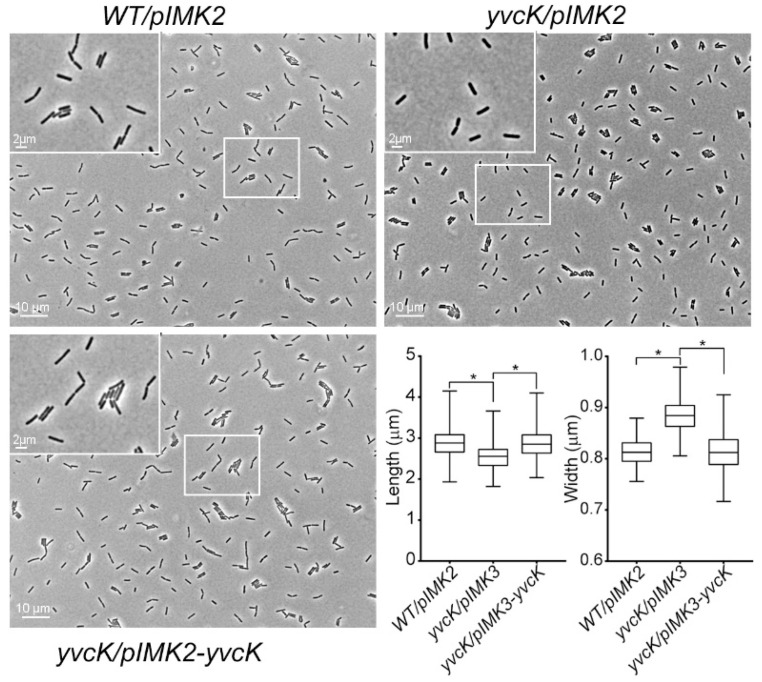
Microscopic cell dimension measurement of *L. monocytogenes* WT and *yvcK::Himar1* mutant strain grown in BHI to exponential phase (OD_600_ = 1) without *t*-CIN (but containing 1 mM IPTG). The cell width and length were analysed with MicrobeTracker software [24] and are depicted in the box and whisker plots (displaying Min and Max at the whiskers, 25 to 75 percentiles at the box and median in the centre line). *n* = 300. *, significant difference at *p* < 0.0001 by two-tailed Student’s *t* test.

In BHI broth with 2 mM *t*-CIN, the *yvcK::Himar1* mutant showed attenuated growth, with a significantly extended lag phase compared to WT (48.2 h vs. 8.9 h) (Figure 2B,C). In addition, the mutant also exhibited a lower OD_max_ and growth rate. Complementation with the wild-type *yvcK* allele (*yvcK/pIMK3-yvcK*), but not with the two downstream genes of *yvcK* (data not shown), completely restored the phenotype, confirming the role of YvcK in *t*-CIN tolerance. Time-lapse microscopy confirmed the attenuated growth of the WT strain in the presence of 1 mM *t*-CIN and revealed a filamentous shape with swellings at the pole of some bacteria (Figure 3, 15 h). More pronounced pole bulging and cell lysis were observed for the *yvcK* mutant (Figure 3, bottom panel, 15 h and 20 h). This bulging cell shape and cell lysis indicates a severe disruption of cell wall integrity, and can explain the attenuated growth of the *yvcK* mutant in the presence of *t*-CIN. Although the *yvcK* mutant grew almost like a WT strain in the absence of *t*-CIN (Figure 2B,C), its cells at late log phase (OD_600_ = 1 by a cell density meter) are on average 0.07 µm (*p* < 0.05) thicker and 0.36 µm (*p* < 0.05) shorter than those of the WT strain (Figure 4), indicating that YvcK might play a role in cell wall biosynthesis or regulation of *L. monocytogenes*. Normal cell shape was restored upon complementation with the wild-type *yvcK* allele (Figure 4).

### 3.2. Suppressor Mutations Reverse the Sensitivity of yvcK::Himar1 to t-CIN

**Figure 5:**
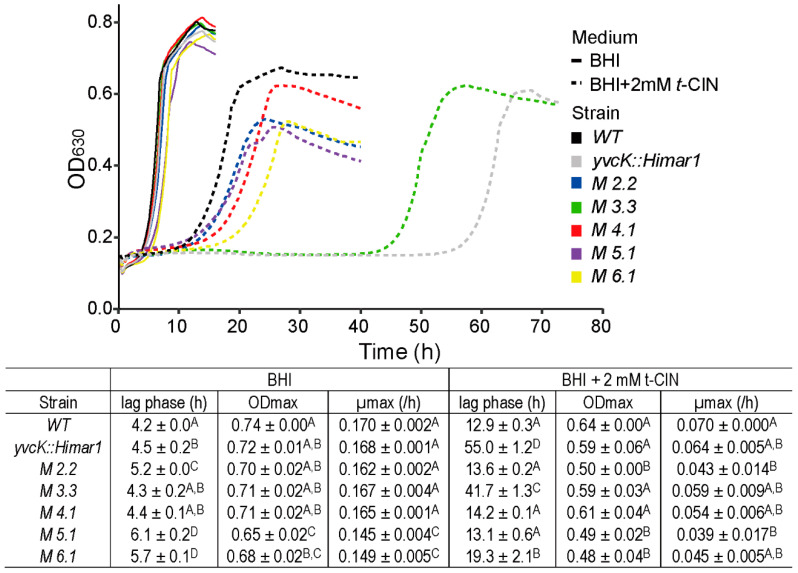
Growth curves and parameters of suppression mutants which partially restore the tolerance of *yvcK::Himar1* to *t*-CIN, in BHI and BHI with 2 mM *t*-CIN. Growth curves represent the average of measurements of three independent cultures. The lag phases (λ), maximum growth rates (µmax) and maximum optical densities (ODmax) are shown in the table as mean ± SD; *n* = 3. Values followed by a common letter are not significantly different at the 5% level of significance.

**Figure 6:**
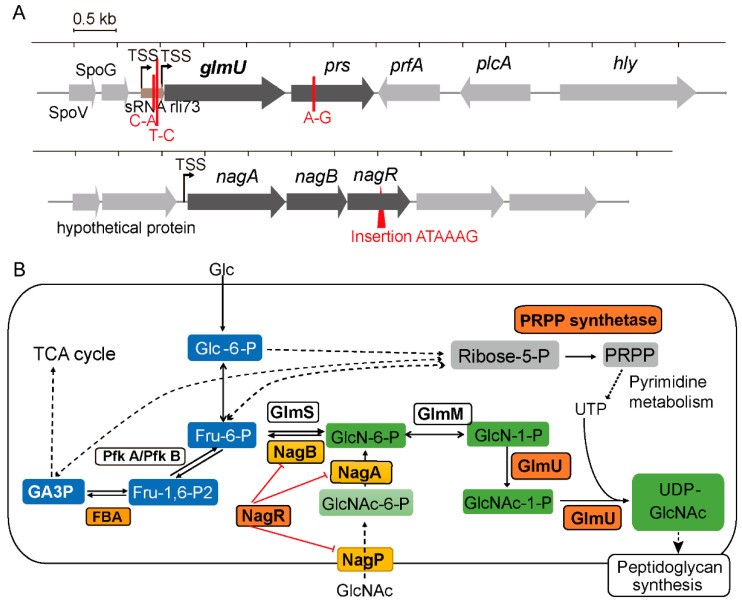
(**A**) Locations of *yvcK* suppression mutations in the *glmU-prs* and *nagABR* operon. Single nucleotide variations are depicted as red bars and the in-frame insertion in *nagR* as a red triangle. Transcription start sites (TSSs) [25] of operons are indicated with a black arrow. (**B**) Scheme of the UDP-GlcNAc biosynthesis pathway with indication of the functions of GlmU and NagR [15,28]. Proteins affected by suppression mutations identified in this work are colored in orange. NagR-suppressed proteins are colored yellow. Intermediates of the glycolysis, the UDP-GlcNAc pathway and the pentose phosphate pathway are shown in blue, green and grey, respectively. PRPP, phospho-alpha-D-ribosyl-1-pyrophosphate; PRPP synthetase, ribose-phosphate pyrophosphokinase; Pfk, phosphofructokinase; and FBA, fructose-bisphosphate aldolase.

To gain insight in the role of YvcK in *t*-CIN tolerance, an evolutionary experiment was performed as depicted in Figure 1, and several isolates with partially restored *t*-CIN tolerance were obtained from the *yvcK::Himar1* mutant. These suppression mutants exhibited lag phases intermediate to those of the *yvcK* mutant and the WT strain when grown in BHI with 2 mM *t*-CIN (Figure 5). In contrast, no strains with restored *t*-CIN tolerance were isolated from the control culture in BHI. Five independent suppressor mutants were subjected to WGS analysis and mutations they have incurred compared to their parental *yvcK::Himar1* strain are listed in Table 3. Two suppression mutants (*M 3.3* and *M 4.1*) had a point mutation immediate upstream of the *glmU-prs* operon, in a region reported to encode the small RNA rli73 [25] (Figure 6A). GlmU is a bifunctional protein, whose C-terminal and N-terminal domain catalyze the sequential conversion of glucosamine-1-phosphate (GlcN-1-P) to GlcNAc-1-P and then to UDP-GlcNAc [15] (Figure 6B). Moreover, *M 4.1* had an additional point mutation causing an amino acid replacement in *fbaA*, which encodes a class II fructose-bisphosphate aldolase. This protein catalyzes the reversible conversion of fructose-1,6-bisphosphate to glyceraldehyde-3-phosphate and dihydroxyacetone phosphate in the glycolysis and gluconeogenesis pathways [26]. Within the *glmU-prs* operon, a point mutation was also found in the coding region of *prs* in suppression mutant *M 6.1* (Figure 6A) (Table 3). The *prs* gene encodes the ribose-phosphate pyrophosphokinase which converts ribose-5-phosphate (Ribose-5-P) into phosphoribosyl pyrophosphate (PRPP), an essential reaction connecting the pentose phosphate pathway with biosynthesis pathways of nucleotides as well as some amino acids and other compounds [27].

Another mutation directly linked to the biosynthesis of UDP-GlcNAc is the 6 bp in-frame insertion in *nagR* in mutant *M 2.2* (Table 3) (Figure 6A), whose product functions as the repressor for GlcNAc utilization (Figure 6B) [28,29,30]. As depicted in Figure 6, *nagR* forms an operon with *nagA* and *nagB* that is under direct control of NagR. Among all the isolates from the evolution experiment, this suppression mutant exhibited the strongest reversion of *t*-CIN sensitivity of the *yvcK* mutant (lag phase from 56 h to 13.6 h), with a lag phase approaching to that of the WT strain (Figure 5). Finally, the growth of mutant *M 5.1*, which has a 6 bp in-frame deletion in *rpoA,* encoding the RNA polymerase subunit alpha, resembled the growth of mutant *M 2.2* in the presence of 2 mM *t*-CIN. However, how the mutated RpoA affects transcription and whether it has a specific impact on the UDP-GlcNAc biosynthesis pathway is unclear and was not further investigated here.

### 3.3. GlcNAc Supplementation Reverses the t-CIN Sensitivity of the yvcK::Himar1 Mutant

**Figure 7:**
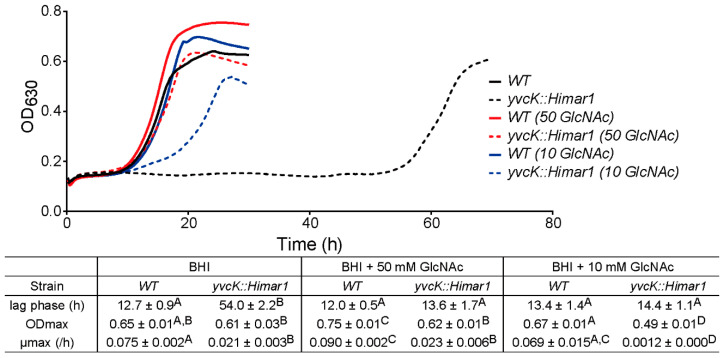
Growth curves and parameters of *L. monocytogenes* WT and *yvcK::Himar1* in BHI broth with 2 mM *t*-CIN, and with or without 50 mM or 10 mM GlcNAc supplementation. Growth curves represent the average of three independent cultures. The lag phases (λ), maximum growth rates (µmax) and maximum optical densities (ODmax) are shown in the table as mean ± SD; *n* = 3. Values followed by a common letter are not significantly different at the 5% level of significance.

Since several of the identified suppression mutations were linked to the biosynthesis of UDP-GlcNAc, we tested whether supplementation of the growth medium with GlcNAc could suppress the growth defect of the *yvcK* mutant in the presence of *t*-CIN. Depending on the concentration, GlcNAc indeed partially (10 mM) or completely (50 mM) reduced the lag phase of the *yvcK* mutant to WT level (Figure 7). The growth of the WT strain in BHI with 2 mM *t*-CIN was also slightly improved by 50 mM GlcNAc, with a slightly higher growth rate and ODmax. The ability of GlcNAc to suppress the *t*-CIN sensitivity of the *yvcK* mutant is consistent with the idea that YvcK regulates UDP-GlcNAc biosynthesis in *L. monocytogenes*. Microscopy also revealed a reduced cell lysis of the *yvcK* mutant in the presence of 1 mM *t*-CIN upon GlcNAc supplementation, although the cells retained their characteristic shape deformation both with and without *t*-CIN (Figures S1 and S2).

The catabolism of GlcNAc is well elucidated in *B. subtilis* [28]. The uptake of GlcNAc into the bacteria is mediated by the GlcNAc-specific phosphoenolpyruvate phosphotransferase system (PTS) protein NagP, which concomitantly phosphorylates GlcNAc to GlcNAc-6-phosphate (GlcNAc-6-P) [28,31] (Figure 6B). GlcNAc-6-P is then converted to GlcN-6-phosphate (GlcN-6-P) by the GlcNAc-6-P deacetylase NagA [28,32]. GlcN-6-P can either be converted to Fru-6-P by GlcN-6-P deaminase NagB [33] or feed into the UDP-GlcNAc biosynthesis pathway [28]. The capacity of GlcNAc to suppress the *t*-CIN sensitivity of the *yvcK* mutant may implicate an insufficient availability of substrate (GlcN-6-P) for UDP-GlcNAc biosynthesis in this mutant under these conditions. 

### 3.4. Overexpression of UDP-GlcNAc Biosynthetic Enzymes Reduce the t-CIN Sensitivity of the yvcK Mutant

**Figure 8:**
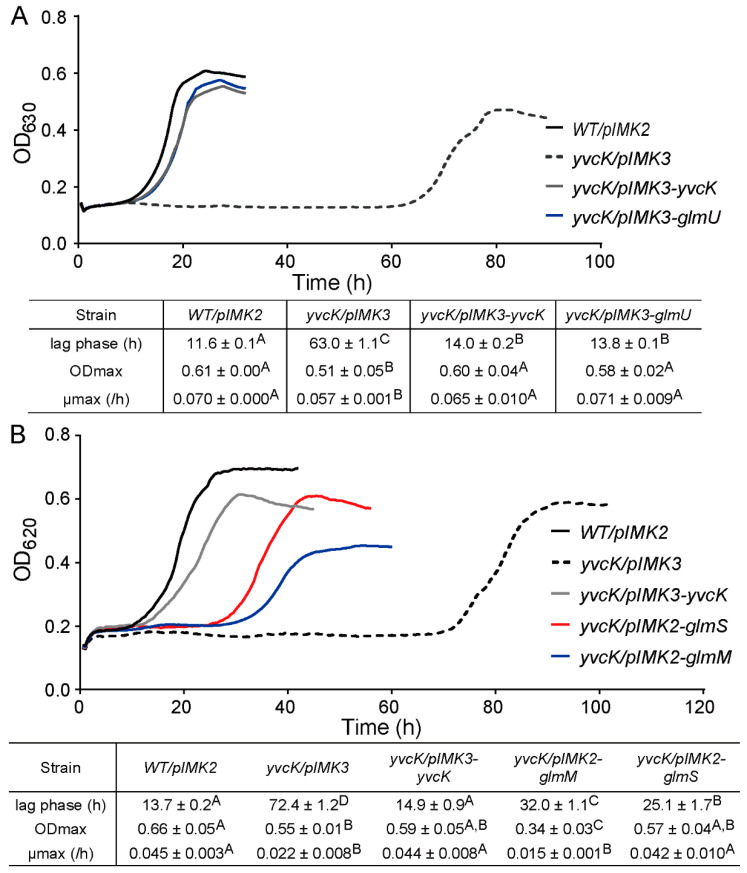
Effect of overproduction of UDP-GlcNAc biosynthetic enzymes on *t*-CIN sensitivity of the *yvcK* mutant. (**A**) Expression of *glmU* on the pIMK3 plasmid was induced by 1 mM IPTG in the *yvcK::Himar1* strain and growth in BHI with 2 mM *t*-CIN and 1 mM IPTG was monitored at 30 °C by measuring OD_630_. (**B**) *glmS* and *glmM* were constitutively expressed from the pIMK2 plasmid in the *yvcK::Himar1* strain and growth curves in BHI with 2 mM *t*-CIN were monitored at 30 °C by measuring OD_620_. The curves represent the average of three independent cultures. The lag phases (λ), maximum growth rates (µmax) and maximum optical densities (ODmax) are specified in the tables as mean ± SD; *n* = 3. Values followed by a common letter are not significantly different at the 5% level of significance.

As aforementioned, two suppression mutations were found immediate upstream of *glmU-prs* operon, in a region demonstrated by transcriptome analysis to encode a small RNA (sRNA) [25] (Figure 6A). Since provision of GlcNAc restored the attenuated growth of the *yvcK* mutant, we hypothesize that transcription of *glmU-prs* is affected by these mutations, leading to increased UDP-GlcNAc biosynthesis. To test whether increased expression of UDP-GlcNAc biosynthetic enzymes suppresses the *t*-CIN sensitivity of the *yvcK* mutant, *glmU*, *glmM* and *glmS* were cloned into pIMK2 or pIMK3 plasmids [21] and introduced into *yvcK* mutant. When expression of *glmU* was induced by IPTG, the sensitivity of *yvcK* mutant to *t*-CIN was effectively restored to almost WT level (Figure 8A). Expression of *glmM and glmS* also restored the *t*-CIN tolerance of the *yvcK* mutant, but only partially (Figure 8B). These results suggest that increased levels of these UDP-GlcNAc biosynthetic enzymes promote the flux of substrate into the UDP-GlcNAc biosynthetic pathway. In contrast, overexpression of these proteins in the WT strain did not further increase *t*-CIN resistance (Figure S3), indicating that the growth-limiting factor of WT in the presence of *t*-CIN is not UDP-GlcNAc homeostasis. 

### 3.5. Complementation with Mutated nagR Allele from Strain M 2.2 Partially Restores Sensitivity of yvcK::Himar1 to t-CIN

**Figure 9:**
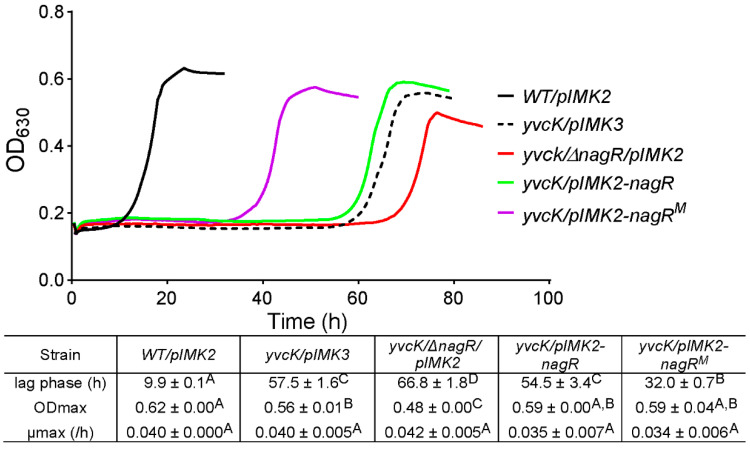
Growth curves and parameters of *yvcK::Himar1* mutant with *nagR* or *nagR^M^* allele expressed from integrated pIMK2 plasmid (designated as *yvcK/pIMK2-nagR* and *yvcK/pIMK2-nagR^M^*, respectively). Bacterial growth in BHI supplemented with 2 mM *t*-CIN was monitored at 30 °C by measuring OD_630_. In addition, the *nagR* deletion strain (*yvcK/ΔnagR/pIMK2*) was included in the growth assay. All curves represent mean values of three independent cultures. Lag phases (λ), maximum growth rates (μmax) and maximum optical densities (ODmax) are listed in the table and represented as mean ± SD, *n* = 3. Values followed by a common letter are not significantly different at the 5% level of significance.

Since NagR acts as the repressor of GlcNAc utilization genes, we anticipated that deletion of *nagR* might cure the *t*-CIN sensitivity of the *yvcK* mutant. However, unexpectedly, the opposite effect was observed, with a further lag time extension by several hours (Figure 9. Medium supplementation with GlcNAc also did not suppress *t*-CIN sensitivity of the *yvcK*-*nagR* double mutant, unlike what was the case for the *yvcK* mutant (Figure S4). A possible explanation of this behavior is the derepression of *nagB* expression in absence of NagR [28], which routes the incoming GlcNAc to glycolysis rather than to UDP-GlcNAc synthesis (Figure 6B). Likewise, in *B. subtilis*, GlcNAc supplementation only slightly lowered the sensitivity of a *yvcK* null mutant to the beta-lactam antibiotic cefuroxime, while disruption of the route from GlcNAc to Fru-6-P by disruption of *nagB* reduced the sensitivity of this mutant to WT level [13]. Interestingly, overexpression of the mutated *nagR^M^* allele (the mutated *nagR* from strain *M 2.2*) in the *yvcK* mutant (*yvcK/pIMK2-nagR^M^)* significantly reduced the sensitivity of the bacteria to *t*-CIN, with a reduction in the lag time from 58 h to 32 h (Figure 9). However, the strain remained more sensitive than the suppression mutant *M 2.2* in which the *nagR^M^* mutation was identified (Figure 5). Overexpression of wild-type *nagR*, in contrast, showed no impact on the *t*-CIN sensitivity of the *yvcK* mutant (Figure 9). Also, neither inactivation of NagR nor overproduction of NagR or NagR^M^ affected the resistance of WT bacteria to *t*-CIN (Figure S3). Altogether, we suspect that the mutation in NagR^M^ modulates its repression of target genes.

## 4. Discussion

In this work, we investigated a previously isolated *t*-CIN hypersensitive *yvcK::Himar1* mutant and link the vulnerability of the mutant to *t*-CIN to elevated cell lysis invoked by impaired cell wall integrity (Figure 4). Evolutionary experiments led to the identification of suppression mutations in genes involved in the biosynthesis of the major peptidoglycan precursor UDP-GlcNAc (Table 3). Chemical supplementation of GlcNAc restored the attenuated growth of *yvcK* mutant in the presence of *t*-CIN (Figure 7), suggesting an insufficient substrate availability for UDP-GlcNAc biosynthesis in the *yvcK* mutant when grown in the presence of *t*-CIN. This idea was further supported by the observation that overproduction of UDP-GlcNAc biosynthetic enzymes in the *yvcK* mutant fully or partially restored the resistance to *t*-CIN (Figure 8). Together, this collective evidence validates a role of YvcK in UDP-GlcNAc biosynthesis in *L. monocytogenes*. A similar role has been recently proposed for YvcK in *B. subtilis,* but based on different evidence [13]. In *B. subtilis*, suppressor mutations were identified which reversed the sensitivity of a *yvcK* mutant to cefuroxime or the growth defect on gluconeogenic carbon sources, and these mutations were shown to elevate the expression of *glmS* and *glmM* [13]. Moreover, supplementation of GlcNAc also reversed the phenotypes of YvcK deficiency in *B. subtilis* [13]. These results suggest a conservative function of YvcK in UDP-GlcNAc biosynthesis in both bacteria.

In *B. subtilis*, the function of YvcK has been studied in greater detail and depends on the availability of glycolytic carbon sources; a ∆*yvcK* mutant exhibited attenuated growth and altered cell morphology when grown on non-glycolytic carbon sources, but provision of glucose, which drives glycolytic carbon flux and thus generates an elevated level of intracellular glycolytic intermediates, revitalized the growth of the mutant [13,14]. The *L. monocytogenes yvcK* mutant also displayed altered cell morphology when grown in BHI (Figure 4). However, since BHI has a complex nutrient composition and contains 2 g/L added glucose, our data do not allow to clearly assess the role of glycolytic carbon sources on the phenotypes of the *L. monocytogenes yvcK* mutant.

Being an electrophilic and thiol-reactive compound, *t*-CIN is anticipated to induce an intracellular redox disbalance [34,35]. This is indeed reflected by the induction of oxidative-stress-related genes upon *t*-CIN exposure, as demonstrated in *E. coli* [35,36,37]. Maintenance of the bacterial intracellular redox homeostasis depends on various enzymatic antioxidant systems and reducing agents such as glutathione [38,39]. Another critical molecule for the antioxidant defense is NADPH, which fuels the regeneration of glutathione and diverse enzymatic antioxidant systems [39]. The cellular NADPH, in turn, is predominantly replenished via the oxidative pentose phosphate pathway (PPP) into which the glycolytic carbon flux will be rerouted when cells are exposed to oxidative stress [40,41,42]. Thus, *L. monocytogenes* might respond to an oxidative *t*-CIN challenge by driving glycolytic substrates to the oxidative PPP to stabilize the intracellular redox state and alleviate the damage caused by *t*-CIN. Proteomic analysis of *E. coli* treated with a sublethal concentration of *t*-CIN showed that the expression of genes involved in PPP is indeed highly upregulated [35], implying an increased carbon flux to the PPP. While it increases the production of NADPH, this reallocation of carbon flux at the same time decreases the glycolytic production of Fru-6-P, which is the basis of the UDP-GlcNAc biosynthetic reactions (Figure 6B). This mechanism can explain the cell shape deformations induced by *t*-CIN (Figure 3). It also explains the hypersensitivity of the *yvcK* mutant, in view of the role of YvcK to control the carbon flux into the UDP-GlcNAc biosynthesis pathway. However, cell shape deformations could alternatively also be explained by interference of *t*-CIN with cytoskeletal elements [43]. In fact, one specific study has claimed *t*-CIN to inhibit the polymerization of FtsZ protein and cell separation in *E. coli*, thus inducing cell filamentation [8].

Furthermore, *t*-CIN may not only destabilize the overall cellular redox balance, but it may also have one or more specific thiol-containing targets in the pathways mentioned above. For example, it may inhibit the activity of glyceraldehyde 3-phosphate dehydrogenase, a glycolytic enzyme that exhibits sensitivity to electrophilic attack due to its Cys active site [40], and hence block the glycolytic and gluconeogenetic flux. Also GlmS, the first enzyme of the UDP-GlcNAc pathway (Figure 6B), has a Cys active site in its N-terminal glutaminase domain that may be targeted by electrophiles [44,45].

The reaction connecting glycolysis and UDP-GlcNAc biosynthesis is mediated by GlmS [46]. In many Gram-positive bacteria, the intracellular concentration of GlmS is post-transcriptionally regulated by *glmS* ribozyme, a *cis*-regulatory structure in the 5′ untranslated region of *glmS* mRNA which activates the degradation of *glmS* transcript by RNase upon binding to GlcN-6-P [47,48]. This mechanism thus provides feedback inhibition on the production of GlcN-6-P from Fru-6-P by GlmS. Recent work in *B. subtilis* indicated that YvcK provides an additional level of control, by stimulating the activity of GlmS in a UDP-GlcNAc dependent manner [13,16]. When the intracellular UDP-GlcNAc content is high (>0.1 mM), the activation of GlmS by YvcK will be inhibited [16], probably by the binding of UDP-GlcNAc to YvcK [49]. 

Furthermore, YvcK was reported to interact with YvcJ, encoded by the gene immediately upstream of *yvcK*, also in a UDP-GlcNAc concentration-dependent manner [16], but the precise role of YvcJ is still unclear. *L. monocytogenes* also has a *yvcJ* homolog upstream of *yvcK*, whose product shares a 67% identity with YvcJ of *B. subtilis* 168, and thus a similar interaction between YvcK and YvcJ may exist in *L. monocytogenes* as well. YvcJ of both *L. monocytogenes* and *B. subtilis* share high sequence identity with RNase adapter protein RapZ of *E. coli*, which interacts with two small RNAs, GlmY and GlmZ, to regulate the intracellular GlmS concentration in response to the intracellular GlcN-6-P level [50,51]. The exact role of YvcJ in regulating GlmS activity and UDP-GlcNAc biosynthesis in *B. subtilis* and *L. monocytogenes* demands further investigation. 

In *B. subtilis*, the NagR repressor was shown to bind to specific operator sites called *dre*-sites, in the promoter region of *nagP* and the *nagABR* operon [28,52,53]. Upon binding to the *dre*-sites, transcription of downstream genes is blocked by NagR [28]. The binding affinity of NagR is tuned by its interaction with ligands, in particular GlcN-6-P and GlcNAc-6-P [53]. Crystal structure analysis of the NagR-ligand complex showed that the phosphate group ligand is coordinated by multiple residues including Thr90, Ser165, Ile166, Tyr167, Arg 133 and Arg135, all of which are conserved in *L. monocytogenes* Scott A NagR (considering the substitution of the Ile by a Leu residue as conservative). Also five out of seven residues proposed to interact with the sugar moiety of the ligands (Ser88, Phe89, Glu145, Arg 211, Glu222, Ala 224 and Tyr 228) are conserved between *B. subtilis* and *L. monocytogenes* NagR [52,53] (Figure S5). Interestingly, the NagR^M^ mutant allele has an Ile and Lys insertion that interrupts the three consecutive residues (Ser-Leu-Tyr) proposed to interact with the phosphate group. This mutation is therefore likely to modify the interaction of NagR^M^ with its ligands, and thus to modulate the expression of *nagP* and the *nagABR* operon. A reduced ligand affinity would maintain the repressor activity of NagR^M^ at higher ligand concentration, and thus in particular reduce expression of NagB, and thereby favor the synthesis of UDP-GlcNAc over the breakdown of GlcN-6-P (Figure 6B).

Suppressor mutations of *yvcK::Himar1* were also identified in genes whose products do not relate directly to UDP-GlcNAc biosynthesis. One point mutation was located in *prs* (Table 3), encoding PRPP synthetase which catalyzes the reversible conversion of Ribose-5-P to PRPP, thereby connecting the PPP with the biosynthesis of nucleotides [27]. Ribose-5-P is a key metabolite of the PPP and can be produced by the oxidative and nonoxidative part of the pathway [54] (Figure 6B). Fru-6-P and glyceraldehyde-3-phosphate can be reversibly converted to Ribose-5-P (and Xylulose-5-P) through different steps of the nonoxidative PPP reactions without NADPH generation [54]. In contrast, the unidirectional oxidative PPP reactions oxidize Glu-6-P to Ribulose-5-P (and CO_2_), with generation of NADPH [39,41,42]. Ribulose-5-P is then further converted to Ribose-5-P (and Xylulose-5-P) [54]. As aforementioned, *L. monocytogenes* might divert the glycolytic carbon flux into the oxidative PPP to generate NADPH and counteract the oxidative stress induced by *t*-CIN, and this would be accompanied by the synthesis of Ribose-5-P. If the mutation in *prs* compromises the activity of the PRPP synthetase, this could push more Ribose-5-P through the nonoxidative PPP to produce glyceraldehyde-3-phosphate and Fru-6-P, the substrate for UDP-GlcNAc synthesis. On the other hand, reduced PRPP activity would potentially also limit the biosynthesis of UDP and other nucleotides (Figure 6B), and could in this way also prevent UDP-GlcNAc production, but although mutant *M6.1* shows a mild growth attenuation in BHI, (Figure 5), additional experiments would be required to ascribe this to nucleotide limitation because nucleotides are unlikely to limit growth in this medium. 

Interestingly, two suppressor mutants (*M3.3* and *M4.1*) have a mutation in Rli73, a presumed small RNA immediately upstream of the *glmU-prs* operon. The function of Rli73 has not been identified, but our result strongly suggest that it may affect expression of the downstream operon. The precise effect remains open to speculation, because both suppressor strains also have a second mutation that can potentially interfere. Of note, mutant *M 4.1* contains a mutated fructose-biphosphate aldolase that could increase the cellular pool of Fru-6-P and thus account for the higher *t*-CIN resistance compared to mutant *M 3.3* (Figure 5). Likewise, suppression mutations of a *B. subtilis yvcK* null mutant were found in genes involved in the glycolysis, PPP or gluconeogenesis when bacteria were grown on nonpreferred carbon sources [13,14]. One suppression mutation was found in the glycolytic gene regulator CggR [14], which represses the transcription of the *gapA* operon encoding five glycolytic enzymes [55]. Suppression mutations were also commonly found in *zwf,* encoding the glucose-6-phosphate dehydrogenase [13,14]. This enzyme catalyzes the conversion of Glu-6-P into gluconate 6-phosphate and connects the glycolysis pathway with the PPP [56]. These suppression mutations might all enrich the cellular Fru-6-P levels and thus the flux into UDP-GlcNAc biosynthesis, thereby alleviating the metabolic defect of a *yvcK* null mutant. 

In conclusion, this study identifies peptidoglycan synthesis, and more specifically biosynthesis of the UDP-GlcNAc precursor, as a pathway that limits the tolerance of *L. monocytogenes* to *t*-CIN, and possibly to thiol-reactive antimicrobials. In addition, the work sheds light on the role of YvcK in diverting glycolytic intermediates into UDP-GlcNAc biosynthesis pathway, especially when the glycolytic intermediate Fru-6-P is running low. However, the precise regulatory activity of YvcK remains ambiguous and needs further investigation. A detailed structural analysis would help to clarify the interaction of YvcK with its suspected target proteins such as GlmS and YvcJ, and the modulation of this interaction by chemical effectors. Given the high conservation of YvcK in Gram-positive bacteria, uncovering its function will improve our understanding of peptidoglycan precursor biosynthesis in a wide variety of pathogens. Since the presence of *t*-CIN significantly attenuates the growth and morphology of the *yvcK* mutant, we anticipate that this compound and other thiol-reactive essential oil compounds may act synergistically with antibiotics targeting peptidoglycan precursor biosynthesis.

## Figures and Tables

**Table 1:** Strains and plasmids used in this work. The superscript “R” after antibiotics denotes resistance.

Bacterial Species	Designation in This Work	Description	Reference
*L. monocytogenes*	WT	wild-type strain Scott A; WGS accession number at NCBI: CM001159	[17]
	*WT/pIMK2*	WT with pIMK2 integrated, Km^R^	
	*yvcK::Himar1*	Transposon insertion in *yvcK*, Em^R^	
	*yvcK/pIMK3*	*yvcK::Himar1* with pIMK3 integrated, Km^R^ Em^R^	
	*yvcK/pIMK3-yvcK*	*yvcK::Himar1* with pIMK3-yvcK integrated, Km^R^ Em^R^	
	*yvcK/pIMK3-glmU*	*yvcK::Himar1* with pIMK3-glmU integrated, Km^R^ Em^R^	
	*yvcK/pIMK2-glmM*	*yvcK::Himar1* with pIMK2-glmM integrated, Km^R^ Em^R^	
	*yvcK/pIMK2-glmS*	*yvcK::Himar1* with pIMK2-glmS integrated, Km^R^ Em^R^	
	*yvcK::Himar1 ΔnagR*	In-frame deletion of *nagR* in *yvcK::Himar1* strain, Em^R^	
	*yvcK::Himar1 ΔnagR/pIMK2*	*yvcK::Himar1 ΔnagR* with pIMK2 integrated, Km^R^ Em^R^	
	*yvcK/pIMK2-nagR*	*yvcK::Himar1* with pIMK2-nagR (WT allele) integrated, Km^R^ Em^R^	
	*yvcK/pIMK2-nagR^M^*	*yvcK::Himar1* with pIMK2-nagR (mutated allele from *M 2.2* suppression mutant) integrated, Km^R^ Em^R^	
*E. coli*	S17-1 λpir	Donor strain for plasmid conjugation	[19]
	DH5-α	Host strain for plasmid constructs	[18]
Plasmids	Description		Reference
pIMK2	Site-specific listerial integrative vector, Phelp constitutive promoter, 6.2 kb, Km^R^		[21]
pIMK3	Site-specific listerial integrative vector, Phelp IPTG inducible promoter, 7.5 kb, Km^R^		[21]
pIMK3-yvcK	pIMK3 with *yvcK* gene (locus tag: LMOSA_4390) from Scott A		
pIMK3-glmU	pIMK3 with *glmU* gene (locus tag: LMOSA_10910) from Scott A		
pIMK2-glmM	pIMK2 with *glmM* gene (locus tag: LMOSA_1060) from Scott A		
pIMK2-glmS	pIMK2 with *glmS* gene (locus tag: LMOSA_16310) from Scott A		
pIMK2-nagR	pIMK2 with *nagR* gene (locus tag: LMOSA_18480) from Scott A		
pIMK2-nagR^M^	pIMK2 with mutated *nagR^M^* gene from *M 2.2* suppression mutant strain		
pKSV7-oriT	Temperature-sensitive shuttle vector for making gene deletion in *L. monocytogenes*, 6742 bp, Amp^R^, Cm^R^		[22,23]
pKSV7-oriT--∆nagR	pKSV7-oriT with 1 kb flanking fragments upstream and downstream of *nagR* inserted		

**Table 2:** Primers used in this work.

Primer	Sequence (5′–3′) *	Reference
yvcK_NcoI	GCATCCATGGGAAAAAAGGAAATGAAACC	
yvcK_SalI	CACTGTCGACTCACTCCTTTTCAATAG	
glmU_NcoI	ATATCCATGGAATCAAAACGATATGCTGTAGTGC	
glmU_SalI	ATATGTCGACTTATTTACCGTGATTCAAATGTTTTGC	
glmM_NcoI	ATATATCCATGGGTAAATATTTTGGTACGGATGGAGT	
glmM_SalI	ATATATGTCGACTGTTGTTTTAATCGTTAAGTGCCAT	
glmS_NcoI	ATATATCCATGGAATGTGGAATCGTTGGATATATTGGAA	
glmS_SalI	ATATATGTCGACTTATTCTACTGTGACACTTTTTGCTA	
nagR-KO-A	ATATGGTACCGGCTGGTAAGGATGCAGATTT	
nagR-KO-B	CATTTTCCCGCCCTCTTCTT	
nagR-KO-C	AAGAAGAGGGCGGGAAAATGATGAAACTCAGGCAGATTACAACA	
nagR-KO-D	ATATCTGCAGCAAGTGTCCCAGCGATTAACA	
nagR_BspHI	ATATATTCATGATCGATAAACAATCAGGAATAC	
nagR_SalI	ATATATCTGCAGTTATTGTTTAATCCTAGCTACAAATTGAA	
pIMK_FW	GAGTCAGTGAGCGAGGAAGC	[7]
pIMK_REV	CCTATCACCTCAAATGGTTCG	[7]
NC16(II)	GTCAAAACATACGCTCTTATCGATTC	
pKSV7-CK-F	TAGCTCACTCATTAGGCAC	
pKSV7-CK-R	TAAGGAGAAAATACCGCATCA	

* Restriction sites are underlined: NcoI (CCATGG), SalI (GTCGAC), BspHI (TCATGA), KpnI (GGTACC) and PstI (CTGCAG).

**Table 3:** Mutations identified by WGS in the suppression mutants that partially restore *t*-CIN tolerance of *yvcK::Himar1*.

Strain	Mutations	Coding Region Change	Location in Gene	Affected Gene	Encoded Product
*M* 3.3	C-A		−108 b from *glmU* start codon	*rli73*	Small RNA rli73
	T-C		243 b downstream of peroxide-responsive repressor gene *perR*		Noncoding region
*M* 6.1	A-G	Y97C	+290 b from *prs* start codon	*prs*	Ribose-phosphate pyrophosphokinase
*M* 2.2	In frame insertion ATAAAG	Insertion of I K between L165-Y166	between +495 b–+496 b of *nagR* start codon	*nagR*	Transcriptional regulator NagR
*M* 4.1	T-C		−111 bp from *glmU* start codon	*rli73*	Small RNA rli73
	C-A	W239L	+716 bp from *fbaA* start codon	*fbaA*	Fructose-bisphosphate aldolase
*M* 5.1	In frame deletion ACCACG	Deletion of R144G145	from +430 b to +435 b of *rpoA*	*rpoA*	DNA-directed RNA polymerase subunit alpha

## Data Availability

Detail of data will be provided on request.

## Supplementary Materials

The following are available online at https://www.mdpi.com/article/10.3390/foods10071666/s1, Figure S1: Time-lapse observation of *yvcK::Himar1* in BHI supplemented with 1 mM *t*-CIN and with or without 10 mM GlcNAc at 30 °C., Figure S2: Phase contrast microscopy of WT and *yvcK::Himar1* in BHI with and without 50 mM GlcNAc (in absence of *t*-CIN)., Figure S3. Effect of overexpression of enzymes involved in GlcNAc metabolism on *t*-CIN sensitivity of WT *L. monocytogenes*., Figure S4. Growth curves of *yvcK::Himar1/**∆nagR* at 30 °C in BHI with 2 mM *t*-CIN, with or without 10 mM GlcNAc. Figure S5., Alignment of NagR amino acid sequences from *L. monocytogenes* Scott A (GenBank accession no.: EGJ24460.1) and *B. subtilis* 168 (GenBank accession no.: WP_003228089.1).
