# Supplementary material for: The Natural Antimicrobial trans-Cinnamaldehyde Interferes with UDP-N-Acetylglucosamine Biosynthesis and Cell Wall Homeostasis in Listeria monocytogenes
Source: Foods. 2021 Jul 20;10(7):1666. doi: 10.3390/foods10071666 (PMC8307235; doi:10.3390/foods10071666)
Supplement: Supplementary file 1 [file foods-10-01666-s001.zip › foods-1279560-supplementary.pdf]

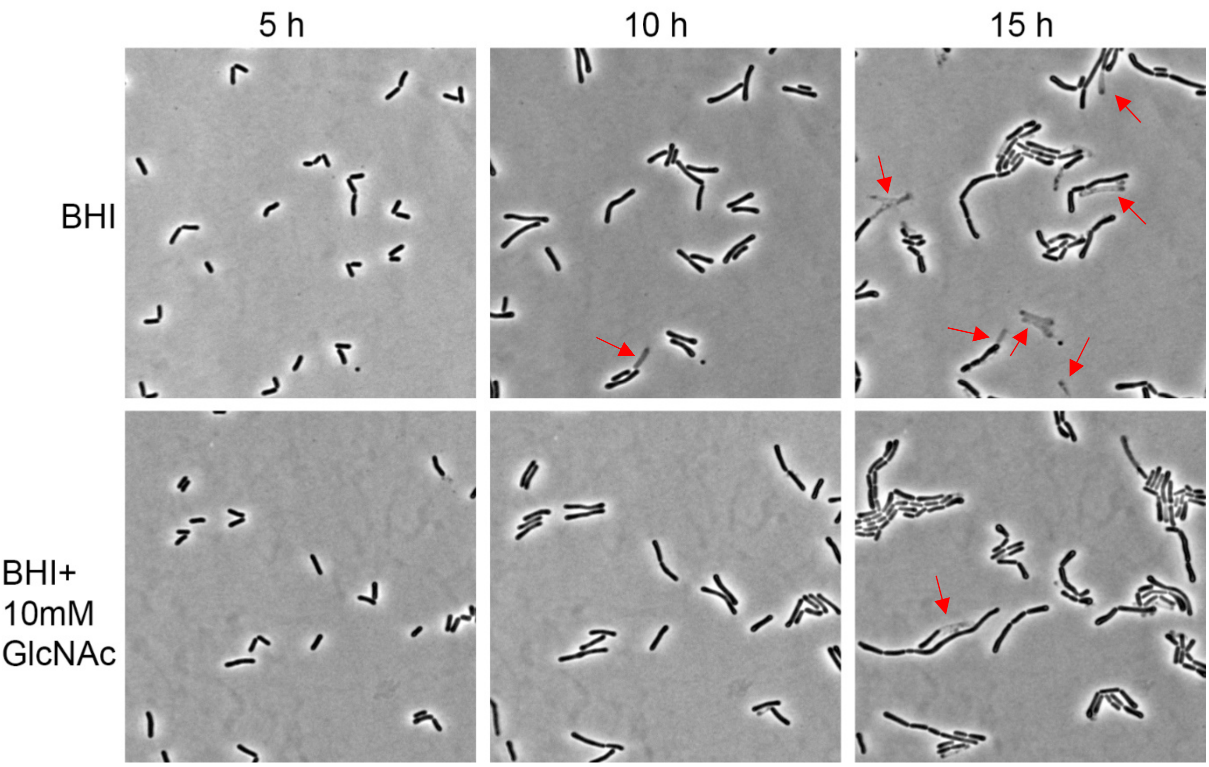

Figure S1. Time-lapse observation of *yvcK::Himar1* cells in BHI supplemented with 1 mM *t*-CIN and with or without 10 mM GlcNAc at 30°C. Phase contrast images of a representative field at 5 h, 10 h, and 15 h are presented. The red arrow indicates cell lysis of *yvcK::Himar1*. Addition of GlcNAc (bottom series) clearly reduced the occurrence of cell lysis at 15 h.

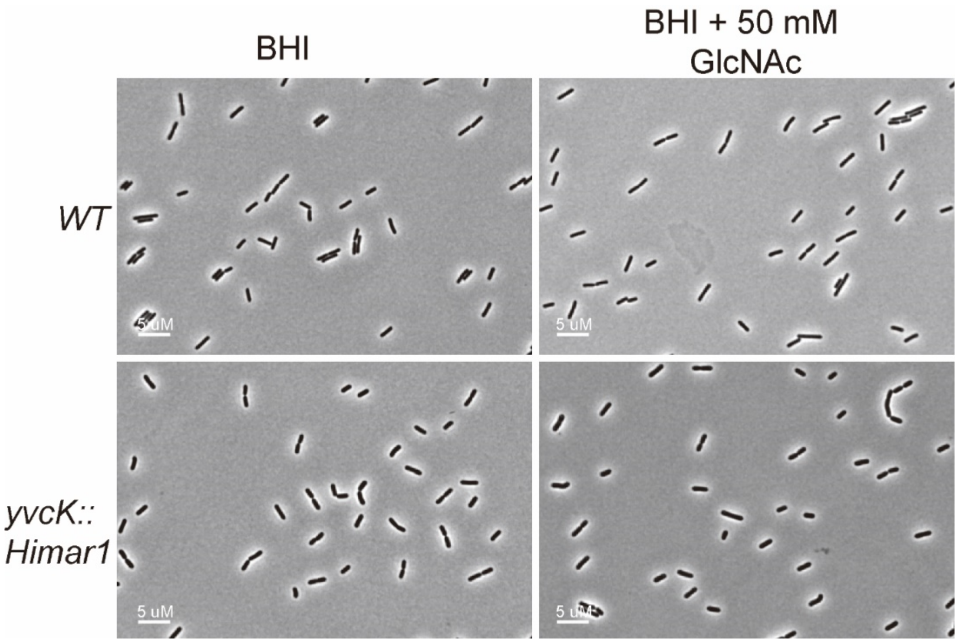

Figure S2. Phase contrast microscopy of WT and *yvcK::Himar1* in BHI with and without 50 mM GlcNAc (in absence of *t*-CIN). Cultures were grown at 30°C till OD_{600} = 1.0. Images are representative of three biological replicates.

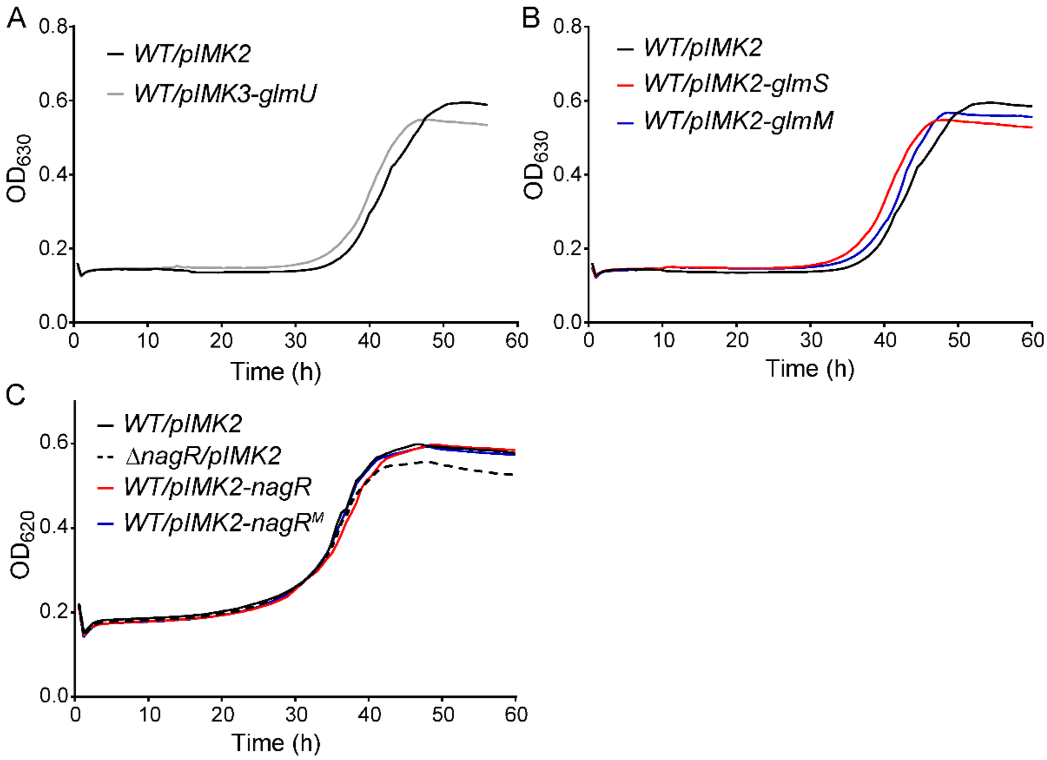

Figure S3. Effect of overexpression of enzymes involved in GlcNAc metabolism on *t*-CIN sensitivity of WT *L. monocytogenes*. A Δ*nagR* strain was included for comparison. Where pIMK2 was used, (over)expression was constitutive, while in case pIMK3 was used, it was induced by 1 mM IPTG. Bacterial growth was monitored by OD_{620} in BHI with 3 mM *t*-CIN at 30°C. All curves represent the average of three independent cultures. The standard deviation is omitted for clarity.

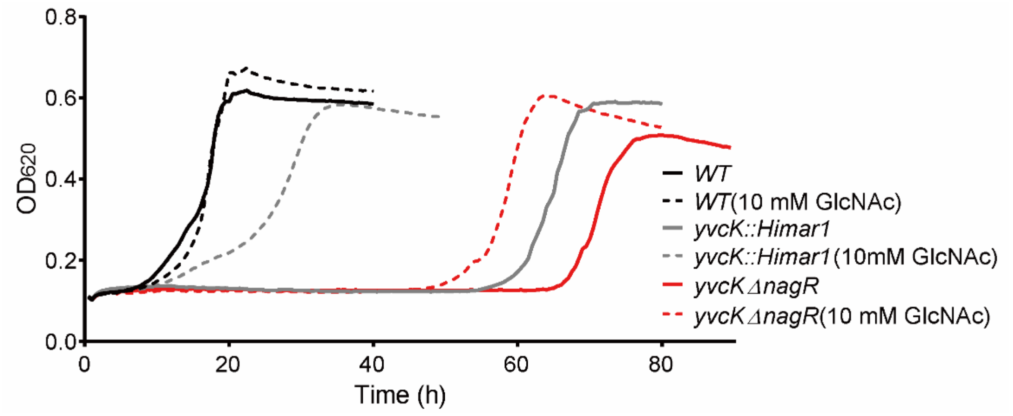

Figure S4. Growth curves of *yvcK::Himar1*/*ΔnagR* at 30°C in BHI with 2 mM *t*-CIN, with or without 10 mM GlcNAc. Curves represent the average of three independent cultures. The standard deviation is omitted for clarity. Supplementation of GlcNAc almost fully restored the tolerance of *yvcK::Himar1* to *t*-CIN, but only slightly reduced the sensitivity of *yvcK::Himar1*/Δ*nagR* to *t*-CIN.

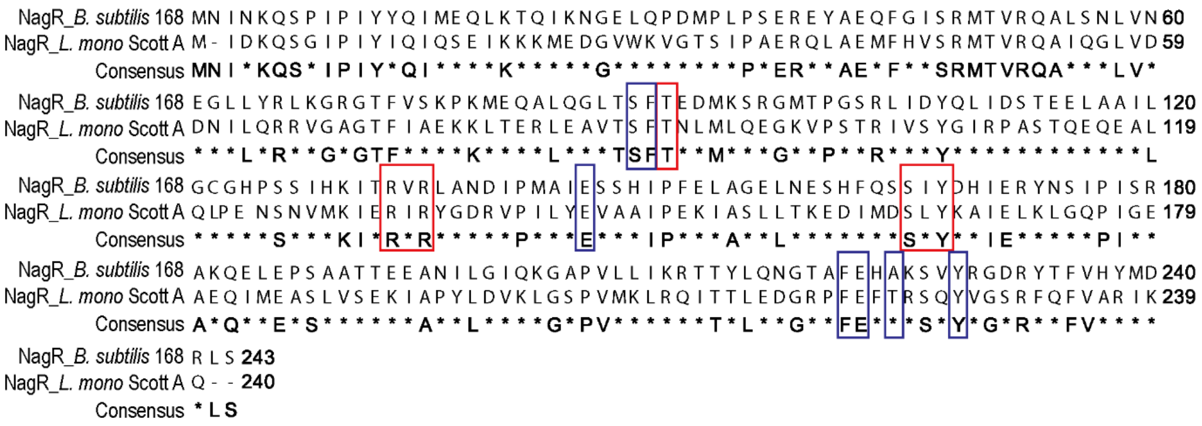

Figure S5. Alignment of NagR amino acid sequences from *L. monocytogenes* Scott A (GenBank accession: EGJ24460.1) and *B. subtilis* 168 (GenBank accession: WP_003228089.1). Both sequences show 35% identity. Residues of *B. subtilis* NagR that are proposed to interact with the phosphate group of the ligands (GlcN-6-P and GlcNAc-6-P), and the corresponding positions in *L. monocytogenes* NagR are highlighted in red boxes. Residues proposed to interact with the sugar moieties of the ligands are shown in blue boxes.
